# Association of physical activity with blood pressure and blood glucose among Malaysian adults: a population-based study

**DOI:** 10.1186/s12889-015-2528-1

**Published:** 2015-12-03

**Authors:** Chien Huey Teh, Ying Ying Chan, Kuang Hock Lim, Chee Cheong Kee, Kuang Kuay Lim, Pei Sien Yeo, Omar Azahadi, Yusoff Fadhli, Aris Tahir, Han Lim Lee, Wasi Ahmad Nazni

**Affiliations:** Institute for Medical Research, Ministry of Health Malaysia, Jalan Pahang, Kuala Lumpur, 50588 Malaysia; Institute for Public Health, Ministry of Health Malaysia, Jalan Bangsar, Kuala Lumpur, 50590 Malaysia

**Keywords:** Hypertension, Diabetes, Malaysia, NHMS, Physical activity

## Abstract

**Background:**

The health-enhancing benefits of physical activity (PA) on hypertension and diabetes have been well documented for decades. This study aimed to determine the association of PA with systolic and diastolic blood pressure as well as blood glucose in the Malaysian adult population.

**Methods:**

Data were extracted from the 2011 National Health and Morbidity Survey (NHMS), a nationally representative, cross-sectional study. A two-stage stratified sampling method was used to select a representative sample of 18,231 Malaysian adults aged 18 years and above. The PA levels of the respondents were categorised as low, moderate or high according to the International Physical Activity Questionnaire (IPAQ)-short form. Blood pressure and fasting blood glucose levels were measured using a digital blood pressure-measuring device and finger-prick test, respectively.

**Results:**

Systolic blood pressure (SBP) level was positively associated with PA level (*p* = 0.02) whilst no significant association was noted between PA level and diastolic blood pressure (DBP). In contrast, respondents with low (adjusted coefficient = 0.17) or moderate (adjusted coefficient = 0.03) level of PA had significantly higher blood glucose level as compared to those who were highly active (*p* = 0.04).

**Conclusions:**

A significant negative association was observed between PA level and blood glucose only. Future studies should employ an objective measurement in estimating PA level in order to elucidate the actual relationship between PA, hypertension and diabetes for the development of effective interventions to combat the increasing burden of premature-mortality and cardiovascular disease-related morbidity in Malaysia.

## Background

Hypertension and diabetes are major and common risk factors for cardiovascular disease. Cardiovascular diseases (CVDs) account for approximately one third of deaths worldwide, with high blood pressure being the leading cause of cardiovascular death in the South East Asia Region. Together with a number of lifestyle risk factors (alcohol use, tobacco use, high body mass index (BMI), high cholesterol, low fruit and vegetable intake and physical activity), high blood pressure and high blood glucose account for 61 % of cardiovascular deaths and more than 75 % of deaths from ischaemic and hypertensive heart disease globally [1].

In Malaysia, the prevalence of hypertension among adults aged 18 years and above has been persistent at 32.2 % in 2006 [2] and 32.7 % in 2011 [3], whereas the prevalence of diabetes has been increasing steadily from 11.6 % in 2006 [2] to 15.2 % in 2011 [3]. The high prevalence of hypertension and diabetes are anticipated to cause an epidemic of cardiovascular disease. The Malaysian Second Burden of Disease and Injury Study (BOD) in 2012 reported that ischaemic heart disease was the top killer and ranked first in the top 10 causes of premature mortality (Years of Life Lost, YLLs) and top 10 based on burden of disease (Disability Adjusted Life Years, DALYs) [4]. Therefore, the implementation of intensive targeted strategies based on scientific evidence to prevent and control these conditions is of paramount importance.

For decades, a plethora of epidemiological studies have demonstrably proven the metabolic and cardiovascular benefits of physical activity (PA), either alone or in conjunction with dietary changes in reducing the risk of hypertension and diabetes [5–7]. Findings from prospective studies [8, 9] and meta-analysis of randomised controlled trials (RCTs) [10, 11] indicated that regular moderate-intensity activity and resistance exercise lowered the blood pressure in both normotensive and hypertensive individuals. Similarly, studies [7, 12, 13] also demonstrated that moderate- and high-intensity PA conferred evident health benefits amongst both non-diabetic and diabetic populations by improving insulin sensitivity and glycaemic control. A RCT reported that the incidence of diabetes among high-risk individuals was reduced by 40–60 % through proper diet control and programmed PA over 3 to 4 years [13].

Nonetheless, despite established evidence of the beneficial relationship between PA, hypertension and diabetes, there is a lack of national data reporting on the association of PA with systolic and diastolic blood pressure as well as blood glucose level among the Malaysian population. Therefore, in the present study, we aimed to determine these associations by analysing data obtained from the National Health and Morbidity Survey (NHMS) 2011 among Malaysian adults aged 18 years and above, while adjusted for other potential confounders. Findings from this study could provide evidence-based information to assist policy-making decisions pertaining to primary prevention and treatment of hypertension and diabetes, as well as interventional strategies.

## Methods

### Study sample

Data were extracted from a cross-sectional multi-ethnic population-based study, the 2011 NHMS, which was conducted from April to July 2011, across 13 states (Penang, Perlis, Kedah, Perak, Selangor, Negeri Sembilan, Melaka, Johor, Kelantan, Terengganu, Pahang, Sabah, Sarawak) and 2 Federal Territories (Kuala Lumpur and Putrajaya) in Malaysia. Samples were selected using a two-stage stratified sampling method, by which the first stage stratification was performed by states and the second stage stratification was performed by urban/rural localities. The urban area is defined as a gazetted administrative area (a carefully mapped area with definite boundaries which had been notified in the government gazette for public information) with adjoining built-up areas of more than 10,000 people while a gazetted administrative area of less than 10,000 people is defined as a rural area. The Malaysian Department of Statistics (DOS) provided the Primary Sampling Units (PSUs) or Enumeration Blocks (EBs) for first stage sampling and Secondary Sampling Units (SSUs) or Living Quarters (LQs) for second stage sampling according to the 2010 census.

A total of 794 EBs (484 urban and 310 rural EBs) were systematically selected from all EBs in Malaysia via a probability-proportional-to-size sampling technique. Subsequently, 12 Living Quarters (LQs) or Secondary Sampling Units (SSUs) were randomly selected from each EB. Finally, all households and eligible household members within the selected LQs were included in the sample. Details of the sampling method and calculation of sample size have been described elsewhere [14]. For the present study, a total of 18,231 eligible respondents (aged 18-year-old and above) were recruited in the study and written consent for participation was obtained before they were interviewed. The study protocol was approved by the Medical Review and Ethics Committee (MREC), Ministry of Health Malaysia.

### Data collection

The data collection was conducted from April 2011 to July 2011 by trained interviewers via face-to-face interviews using a standardised questionnaire; whilst clinical assessment, which included measurement of blood pressure and blood glucose level, was performed by trained staff nurses. To ensure a higher response rate, only selected respondents who were not at home after at least 3 attempted visits were excluded from the survey.

### Physical activity

The International Physical Activity Questionnaire-short form (IPAQ-SF) estimates the overall PA level of an individual in MET-minutes/week by determining the duration (in minutes) and number of days (in one week) of engagement in three specific types of activity (walking, moderate-intensity and high-intensity activities) across a comprehensive set of domains (leisure time, work-related and transport-related physical activities, domestic and gardening activities) in the past seven days. MET or metabolic equivalent is a unit that is used to estimate the amount of oxygen used by the body during physical activity. A MET-minutes/week is computed by multiplying the MET score of an activity (3.3 for walking, 4.0 for moderate-intensity and 8.0 for vigorous-intensity) by the minutes and days (or sessions) of engagement. Respondents were classified into three levels of PA as low, moderate or high according to the cutoff of total MET-minutes/week in each category [15] as follow:

- Category 1 Low

Individuals who do not meet the criteria for Category 2 (moderately active) and 3 (highly active) were considered to have a ‘low’ PA level or were physically inactive.

- Category 2 Moderate
  - 3 or more days of vigorous-intensity activity of at least 20 min per day, or
  - 5 or more days of moderate-intensity activity and/or walking of at least 30 min per day, or
  - 5 or more days of any combination of walking, moderate- or vigorous-intensity activities achieving a minimum total PA of at least 600 MET-minutes/week
- Category 3 High
  - vigorous-intensity activity on at least 3 days achieving a minimum total PA of at least 1500 MET-minutes/week, or
  - 7 or more days of any combination of walking, moderate- or vigorous-intensity activities achieving a minimum total PA of at least 3000 MET-minutes/week.

### Hypertension

Blood pressure level was measured twice in an interval of 10 min by a trained nurse using a calibrated digital blood pressure-measuring device, OMRON HEM-907. This device was evaluated for compliance to both the American Association for the Advancement Medical Instrumentation (AAMI) and British Hypertension Society (BHS) protocols for accuracy for a non-invasive blood pressure-monitoring device using single observer readings [16]. Both known hypertension and undiagnosed hypertension constituted the overall prevalence of hypertension. “Known hypertension” is defined as self-report of being told they have hypertension by a doctor or medical assistant; “undiagnosed hypertension” is defined as self-perceived as non-hypertensive and had a SBP of 140 mmHg or more and/or DBP of 90 mmHg or more [17].

### Diabetes

Respondents were asked to fast for at least 6 h for the measurement of fasting blood glucose level using a validated CardioChek PA Analyser [18] via finger-prick test by trained nurses. Respondents with known diabetes or undiagnosed diabetes constituted the overall prevalence of diabetes. “Known diabetes” is defined as self-report of being told they have diabetes by a doctor or medical assistant; a respondent was classified as having ‘undiagnosed diabetes’ when the respondent was not known to have diabetes and had a fasting capillary blood glucose (FBG) of 6.1 mmol/L or more or non-fasting blood glucose of more than 11.1 mmol/L [19].

### Anthropometric measurements and obesity

SECA bodymeter 206 (SECA, Germany) and TANITA318 digital weighing scale (TANITA, Japan) were used to measure the height and weight of respondents to the nearest 0.1 cm and 0.1 kg, respectively. Anthropometric measurements were taken twice and the average values were used for data analysis. Body mass index (BMI, weight/height^2^ in kg/m^2^) was used to classify respondents into underweight, normal, overweight and obese based on the cut-off points recommended by the World Health Organization (WHO) [20].

### Smoking

Respondents were classified as non-smokers if they answered “*No*” to the question “*Have you ever smoked shisha, cigarettes, cigars and pipes?*”; whilst respondents who answered “*Yes*” to both questions “*Have you ever smoked shisha, cigarettes, cigars and pipes?*” and “*Do you currently smoke?*” were classified as smokers.

### Data analysis

SPSS version 19.0 with add-on complex sample analysis was used to analyse the data after the adjustment for stratification using post-stratified weights. Descriptive statistics were used to illustrate the characteristics of the study population by PA level (low, moderate or high) and socio-demographic variables (gender, locality, age, ethnicity, educational level, household monthly income, employment and marital status). Multivariable linear regression (MLR) or ordinary least square was performed to elucidate the associations of PA levels with SBP, DBP and blood glucose while controlling for gender, locality, age, ethnicity, educational level, household monthly income, employment and marital status, body mass index status and smoking status. Missing data were excluded from analyses and it was ignorable since the NHMS 2011 had taken a 20 % non-response rate into consideration at the sample size calculation stage. All statistical tests were conducted at a 95 % confidence interval (CI).

## Results

Of the 18,231 eligible respondents aged 18 years and above, 99.1, 99.3 and 97.5 % of them responded to the PA, hypertension and diabetes modules of the questionnaire, respectively. The mean age (SD) of the respondents was 42.0 (16.0) years. The characteristics of respondents are provided in Table 1. The population was almost equally composed of men (51.1 %) and women (48.9 %). A large portion of respondents were urban dwellers (73.1 %). Almost half of the Malaysian population was constituted by the Malays, followed by the Chinese (25.4 %), other ethnic groups (Serani, Iban, Kadazan, Dusun, Bidayuh, Melanau and Bumiputras from the State of Sabah and Sarawak, as well as the aborigines, 17.8 %) and Indians (7.0 %). Most of the respondents attained a tertiary level of education (college/university graduates). Men accrued a higher level of high-intensity PA whilst women were engaged more in low- and moderate-intensity activities. Almost 1 in every 3 adults was overweight or hypertensive and 2 in every 13 were diabetic.

**Table 1:** Characteristics of the study population, Malaysia, April-June 2011

Socio-demographics	Male		Female		All	
	*n*	*N* (%)	*n*	*N* (%)	*n*	*N* (%)
	8536	51.1	9695	48.9	18231	100
Locality						
Urban	4874	73.0	5730	73.3	10604	73.1
Rural	3662	27.0	3965	26.7	7627	26.9
Age						
18-24	1445	20.3	1448	20.1	2893	20.2
25-34	1889	26.7	2097	25.7	3986	26.2
35-44	1643	19.9	2000	20.2	3643	20.0
45-54	1620	16.4	1862	16.0	3482	16.2
55-64	1159	10.0	1301	10.2	2460	10.1
65 and above	780	6.7	987	7.8	1767	7.3
Ethnicity						
Malay	4858	48.5	5532	51.0	10390	49.8
Chinese	1666	25.9	1856	25.0	3522	25.4
Indian	651	6.8	808	7.3	1459	7.0
Others	1361	18.8	1499	16.8	2860	17.8
Educational Level						
Primary	2106	22.1	2263	20.8	4369	21.4
Secondary	4091	48.6	4191	45.6	8282	47.1
Tertiary	1825	23.7	2040	23.9	3865	23.8
No formal education	514	5.6	1201	9.7	1715	7.6
Household Income/month^a^						
Less than RM2000	3102	32.3	4038	38.4	7140	35.3
RM2000-RM2999	1526	17.5	1562	16.1	3088	16.8
RM3000-RM3999	1172	14.0	1236	13.2	2408	13.6
At least RM4000	2736	36.2	2859	32.4	5595	34.3
Employment Status						
Government/semi-government	1075	12.3	1073	10.4	2148	11.4
Private	3389	51.0	2308	33.9	5697	42.7
Self-employed	2376	27.4	1243	13.2	3619	20.5
Homemaker/unpaid worker	100	1.3	3094	33.9	3194	17.1
Retiree	789	8.1	949	8.6	1738	8.3
Marital Status						
Single	2356	32.9	1875	24.0	4231	28.6
Married	5954	65.2	6554	65.6	12508	65.4
Widow/Widower/Divorcee	216	1.9	1254	10.4	1470	6.0
BMI Status						
Underweight	638	8.4	639	8.2	1277	8.3
Normal	3833	48.0	3797	46.4	7630	47.2
Overweight	2538	30.9	2615	27.8	5153	29.4
Obese	1021	12.7	1729	17.6	2750	15.1
Smoking Status						
Current smoker	3973	46.9	160	1.9	4133	25
Non-current smoker	4510	53.1	9430	98.1	13940	75
Physical Activity Level						
Low	2581	30.1	3899	40.4	6480	35.2
Moderate	2729	32.5	3898	41.2	6627	36.7
High	3122	37.4	1841	18.4	4963	28.1
Hypertension						
Yes	3130	33.7	3570	31.6	6700	32.7
No	5330	66.3	6068	68.4	11398	67.3
Diabetes						
Yes	1555	15.8	1647	14.5	3202	15.2
No	6754	84.2	7826	85.5	14580	84.8

Abbreviations: *RM* Ringgit Malaysia

^a^1 RM ≈ 0.24 US Dollar

Generally, both simple and multivariable linear regression (MLR) analyses demonstrated that PA level was positively associated with systolic blood pressure (SBP) (*P* < 0.05). However, such association was not significant between PA level and diastolic blood pressure (SDP) in the adjusted MLR analyses (Table 2). In contrast, both simple and multivariable linear regression (MLR) analyses revealed that there was a significant dose response relationship between PA level and blood glucose level (*P* < 0.05). Respondents with low level of PA had significantly higher blood glucose level as compared to those who were moderately or highly active (reference group), and also, those who were moderately active had significantly higher blood glucose level than their highly active counterparts (Table 3).

**Table 2:** Coefficients from multiple linear logistic regression of systolic and diastolic blood pressure on physical activity level

Physical activity level	Systolic blood pressure						Diastolic blood pressure					
	Crude			Adjusted			Crude			Adjusted		
	Coefficient	Standard error	*p-value	Coefficient	Standard error	*p-value	Coefficient	Standard error	*p-value	Coefficient	Standard error	*p*-value
Low (high)	−2.30	0.54	<0.001	−1.35	0.49	0.02	−1.20	0.33	0.001	−0.49	0.33	0.31
Moderate (high)	−1.72	0.51		−0.57	0.48		−0.41	0.32		−0.24	0.32	
n	17,568			14,923			17,567			14,922		

^a^Coefficient values were adjusted for gender, locality, age, ethnicity, educational level, household monthly income, employment and marital status, body mass index status and smoking status

*Significant *p* values at 95 % confidence interval

**Table 3:** Coefficients from multiple linear logistic regression of blood glucose on physical activity level

Physical activity level	Crude			Adjusted		
	Coefficient	Standard error	*p-value	Coefficient^a^	Standard error	*p-value
Low (High)	0.26	0.07	0.002	0.17	0.07	0.04
Moderate (High)	0.12	0.06		0.03	0.06	
n	16,181			13,973		

^a^Coefficient values were adjusted for gender, locality, age, ethnicity, educational level, household monthly income, employment and marital status, body mass index status and smoking status

*Significant *p* values at 95 % confidence interval

## Discussion

This is the first national, multi-ethnic study to cross-sectionally examine the effects of PA against SBP, DBP and blood glucose among Malaysians aged 18 years and above, while controlling for other potential confounders. The present findings demonstrated a positive association between physical activity and systolic blood pressure. However, observations from several well-controlled cohort studies [8, 9] and randomised control trials (RCTs) [10, 11] had proven unequivocally an independent, negative association between PA and blood pressure.

One of the plausible explanations for the above findings could be due to differential misclassification, in which either the hypertensive respondents were differentially misclassified as having high PA or those with low PA were differentially misclassified as non-hypertensive. However, the latter (misclassification of hypertensive status) was unlikely as blood pressure of respondents were measured twice at a 10-min interval using calibrated devices, OMRON HEM-907 by trained nurses. Therefore differential misclassification of hypertensive respondents as highly active, or normal respondents as lowly active could occurred as a result of information bias as the use of IPAQ in the estimation of PA level was rather subjective and subjected to recall bias and tendency of respondents to report a socially desirable response, which may lead to an over-estimation of PA. Lee and his colleagues who performed a systematic review on the validation of IPAQ-SF with objective measurements stated that IPAQ-SF typically overestimated physical activity by an average of 84 % [21]. Therefore, the possible presence of differential misclassification of PA due to poor PA ascertainment in attenuating the real effect of PA against blood pressure should not be overlooked. In addition, more than three-quarters of respondents with known hypertension claimed that they were on oral anti-hypertensive drugs within the past 2 weeks of the interview date (data not shown), and this may again attenuate the beneficial effect of PA against SBP. Furthermore, the cross-sectional nature of the present study had also limited the inference of a direct causal relationship between PA and blood pressure. Therefore, only a properly conducted prospective cohort on healthy individuals [22] or a RCT which employs objective measurement for PA, such as accelerometer, pedometer or doubly labelled water [21], could conclusively determine the causal as well as dose–response relationship between PA level and blood pressure.

In contrast, a significant negative association was observed between PA and blood glucose among Malaysian adults. The present findings were consistent with those reported from other cross-sectional studies [6, 7, 12] and were corroborated by prospective observational studies [8, 23]. Furthermore, an interventional study by Knowler et al. [24] had demonstrated a 58 % (96 % CI: 48–66) reduction of diabetes incidence among the intervention group, which targeted at least a 7 % weight loss and at least 150 min of PA per week, compared to placebo. Additionally, findings from a large cohort study among more than 70,000 healthy women aged 40–65 years in the United States had substantiated the beneficial effects of moderate- and vigorous-intensity PA against diabetes, whereby brisk walking and more vigorous exercise were found to be associated with a 25 % reduction in diabetes incidence [25].

Despite the cross-sectional nature of the study and the use of a subjective instrument (IPAQ) in the estimation of PA level, the large sample size and its representativeness as well as the high response rate were the major strengths of the present study. In addition, underestimations of the prevalence of hypertension and diabetes among the population were unlikely as measurements of blood pressure and fasting blood glucose levels were taken properly by trained nurses using calibrated devices rather than self-reported.

## Conclusions

Generally, a significant dose–response relationship was observed between physical activity and blood glucose, but not between physical activity and blood pressure. Nonetheless, in order to elucidate the true association between physical activity and hypertension or diabetes, the use of an objective instrument for the estimation of the physical activity level is highly recommended for future study.

## Abbreviations

AAMI: American Association for the Advancement Medical Instrumentation
BHS: British Hypertension Society
BMI: body mass index
BOD: burden of disease
CI: confidence interval
CVD: cardiovascular disease
DALYs: disability-adjusted life years
DBP: diastolic blood pressure
DOS: Department of Statistics
EB: enumeration block
FVs: fruits and vegetables
IPAQ: international physical activity questionnaire
LQ: living quarter
MET: metabolic equivalent
MLR: multivariable linear regression
MREC: Medical Review and Ethics Committee
NHMS: National Health and Morbidity Survey
PA: physical activity
PSU: primary sampling unit
RCT: randomised controlled trial
SBP: systolic blood pressure
SD: standard deviation
SSU: secondary sampling unit
WHO: World Health Organisation
YLLs: years of life lost
